# Expression and Characterization of the Novel Gene *fr47* during Freezing in the Wood Frog, *Rana sylvatica*

**DOI:** 10.1155/2015/363912

**Published:** 2015-05-26

**Authors:** Katrina J. Sullivan, Kyle K. Biggar, Kenneth B. Storey

**Affiliations:** Institute of Biochemistry and Department of Biology, Carleton University, 1125 Colonel By Drive, Ottawa, ON, Canada K1S 5B6

## Abstract

The wood frog, *Rana sylvatica*, has numerous adaptations that allow it to survive freezing of up to 65% of its total body water during the winter. Such adaptations have been found to include the expression of novel freeze responsive genes that are thought to be important for adaptation and survival. In this study, the tissue-specific stress responsive expression of one novel gene, *fr47*, was assessed in seven wood frog tissues. In response to freezing, the transcript expression of *fr47* increased significantly in six tissues: heart, lung, liver, skeletal muscle, kidney, and testes. The expression of *fr47* was also strongly upregulated by component stresses of freezing, namely, anoxia and dehydration. A dynamic change in *fr47* expression was also observed during tadpole development, with expression low in embryonic stages (Gosner stages 14–20), increasing through intermediate (stages 26–43) and transformation phases (stages 44-45). These results indicated that *fr47* potentially has a role to play in development and metamorphosis, in addition to freeze, anoxia, and dehydration tolerance. *De novo* analysis of FR47 protein structure revealed a likelihood of membrane associated function and possible GRB2 association. It is hypothesized that this interaction may influence inositol 1,4,5-trisphosphate production, known to increase during wood frog freezing.

## 1. Introduction

Several species of northern woodland frogs display a remarkable ability to endure long term freezing of their body fluids. To date, the physiological, biochemical, and molecular responses to freeze tolerance have been extensively studied in the wood frog,* Rana sylvatica* [1–4]. The initiation of freezing occurs from the nucleation of surface moisture on permeable skin of the frog. Propagation of the ice through the frog will continue until 65% of the total body water is frozen. Importantly, freezing imposes a variety of stresses upon the cells and tissues of the frog that include extensive damage to subcellular architecture and direct physical damage to tissues from ice crystal formation [2]. At a level of the whole organism, a major physiological concern is anoxia (i.e., lack of oxygen) that results through the freezing of the lungs and heart, as well as ischemia (i.e., disruption of blood flow) that occurs once the blood plasma freezes. Another major freeze-associated concern is dehydration, as the formation of extracellular ice from pure water creates hyperosmotic stress on unfrozen cells. Effectively, this causes water to osmotically withdraw from cells, resulting in a sharp decrease in cell volume. The collective consequence of these stresses necessitates specific biochemical and physiological adaptations that ensure the frog will thaw come spring with minimal cellular damage as a result of freezing.

Previous studies have explored freeze-induced changes in gene expression through the use of heterologous screening of cDNA arrays, or by differential display technology [1, 5, 6]. Of particular interest, the screening of cDNA libraries from liver tissue of frozen wood frogs revealed a freeze-induced upregulation of three unique genes, since identified as* li16*,* fr10*, and* fr47* [7–9]. These genes code for freeze responsive novel proteins that show little or no significant homology to any identified gene/protein in current sequence deposited in GenBank.

Currently, the* fr47* is the largest and least understood of these three novel freeze responsive genes [10, 11]. The* fr47* mRNA sequence contains 3678 nucleotides and is translated into a 390-amino-acid protein, with a calculated molecular mass of approximately 46 kDa. A previous analysis of a Kyte-Doolittle hydrophobicity plot of FR47 protein indicated a highly hydrophobic region near the C-terminus of the protein from amino acid positions 350 to 370 [8]. Such hydrophobic regions are common amongst membrane associated proteins and likely represent a transmembrane segment and possible involvement in cell membrane associated cellular processes. The present study presents an in-depth analysis of the expression of* fr47* gene transcripts in multiple organs of wood frogs in response to freezing, anoxia, and dehydration stresses, as well as during development of the tadpole. The data also provides insight into the structure and potential membrane associated function of the FR47 protein in wood frog freeze tolerance.

## 2. Materials and Methods

### 2.1. Animal Treatment and Tissue Preparation

Adult male wood frogs and wood frog eggs were collected from breeding ponds near Bishop's Mills, Ontario, in early spring. Adult frogs were washed in a tetracycline bath and held in containers lined with damp sphagnum moss at 5°C for 1-2 weeks before experiments began. Control adult frogs were sampled from this condition. For freezing exposure, adult frogs were placed into containers lined with damp paper towel and transferred to a −4°C incubator. At this temperature, frogs cool and begin to freeze within 45 to 60 min [12]. After 60 min, temperature was raised to −2.5°C and freeze duration was timed from this point. Frogs were sampled after 24 hr of freezing.

For the anoxia experiments, 5°C acclimated adult frogs were placed in sealed containers (700 mL, 5-6 frogs per jar) that were set in crushed ice and had been previously flushed with nitrogen gas for 15 min. Once containing frogs, containers were then flushed with nitrogen gas for further 20 min and sealed with parafilm. Containers were then placed in a 5°C incubator for 24 hr. After this time, the nitrogen gas line was reconnected and frogs were rapidly sampled.

The adult frogs destined for dehydration experiments were sampled from the 5°C acclimated experimental group of frogs. Containers were placed in an incubator set at 5°C and frogs were allowed to lose water by evaporation. At varying intervals the frogs were quickly reweighed, and the amount of body water lost was calculated using the equation (*M*_*i*_ − *M*_*d*_)/(*M*_*i*_ × %H_2_O), where *M*_*i*_ is the initial mass of the animal, *M*_*d*_ is the mass at each weighing, and %H_2_O is the percentage of total body mass that is water (for control wood frogs, %H_2_O was 80.8 ± 1.2%) [13]. The mean rate of water loss during the experiment was about 0.5% of total body water lost per hr, and animals were sampled immediately after they had lost 40% of their total body water. All frogs were euthanized by pithing and tissues were rapidly excised, immediately frozen in liquid nitrogen, and then stored at −80°C until use.

For wood frog development studies, eggs were housed in a 10-gallon aquarium that contained a mixture of pond water and dechlorinated tap water (~20°C), bubbled with an air stone. Tadpoles were fed shredded lettuce softened by boiling, as well as goldfish flakes daily. Every 2-3 days, all but 1 L of the aquarium water was removed and this was replaced by fresh dechlorinated tap water. The 1 L of aquarium water was retained to supply any microbes essential to the tadpole environment. As the tadpoles developed, they were sampled at various stages and frozen in liquid nitrogen. Sampling occurred at stages defined by Gosner [14] (a) stages 14–20, where the unhatched embryo has developed a neural fold, (b) stages 21–25, where they are hatched tadpoles, (c) stages 26–30, where back limb buds have emerged, (d) stages 31–35, where back limb buds have developed into “paddle” feet (i.e., no toes), (e) stages 36–41, where back limbs have developed individual toes, (f) stages 42-43, where a front limb has developed, and (g) stages 44-45, where the tail is almost completely absorbed and appears as a bud.

### 2.2. RNA Preparation and cDNA Synthesis

Frozen tissues were weighed (100 mg for heart and hind leg skeletal muscle and 50 mg for all others) and homogenized in 1 mL of TRIzol reagent (Invitrogen; Cat# 15596-026). Following homogenization, 200 *μ*L of chloroform was then added and samples were briefly vortexed. Samples were then centrifuged at 10,000 ×g for 15 min at 4°C. The aqueous supernatant (RNA containing fraction) was removed and stored in a 1.5 mL RNase-free Eppendorf tube. From these samples, RNA was then precipitated with the addition of 500 *μ*L isopropanol and was incubated at 20 min at room temperature. Samples were then centrifuged at 12,000 ×g for 15 min at 4°C. The resulting RNA pellet was washed with 1 mL of 70% ethanol and centrifuged again at 7,500 ×g for 5 min at 4°C. The pellet was then air-dried and resuspended in 25 *μ*L of RNase-free H_2_O. RNA quality was determined based on the ratio of 260/280 nm absorbance and quantified using a UV spectrophotometer at 260 nm.

For cDNA synthesis, an aliquot containing 3 *μ*g of total RNA was diluted with RNase-free H_2_O to a final volume of 10 *μ*L. To each sample, 1 *μ*L of 200 ng/*μ*L* oligo dT* primer (5′-TTTTTTTTTTTTTTTTTTTTTTV-3′) was then added and samples were incubated for 5 min at 65°C, followed by rapid cooling on ice for 1 min. Subsequently, 4 *μ*L of 5x first strand buffer, 2 *μ*L of 100 mM DTT, 1 *μ*L of dNTP mixture (25 mM each), and 1 *μ*L of reverse transcriptase M-MLV enzyme (Sigma Aldrich; Cat# M1302-40KU) were added to each sample, followed by incubation for 45 min at 42°C, and then chilled on ice. All cDNA samples were then serial-diluted and stored at −20°C until use.

### 2.3. Polymerase Chain Reaction Amplification

For polymerase chain reaction (PCR), a mixture of 15 *μ*L sterile RNase-free H_2_O, 5 *μ*L diluted cDNA, 1.25 *μ*L primer mixture, 0.75 *μ*L of 10x PCR buffer (Invitrogen), 1.5 *μ*L of 50 mM MgCl_2_, 0.5 *μ*L of dNTP mixture (25 mM each), and 1 *μ*L of* Taq* polymerase was combined for a total volume of 25 *μ*L. Primers used were designed using the Primer Design Program v.3 (Scientific and Educational Software) based on the sequence for* fr47* (GenBank accession AY100690). Primer sequences for* fr47* and *α*-*tubulin* are listed in Table 1. For amplification, the PCR program used was 95°C for 7 min, followed by 28–35 cycles of 94°C for 1 min, 53°C for 1 min, and 72°C for 1.5 min, and finally 72°C for 10 min.

### 2.4. Computer Modeling

Structural analysis of FR47 was performed by first verifying the existence of solved structures with homologous sequences using BLASTp searches against the PDB database. As there were no significant hits, an alternative modeling procedure was employed by using the threading software I-TASSER [15]. The best ranked model according to I-TASSER TM-score (a metric for measuring the structural similarity of two proteins) resembled the folding of a protein whose structure is described in the PDB file 3QXY.pdb (TM-score of 0.874, where a value of 1 indicates a perfect match). The top scoring FR47 model was protonated and optimized by energy minimization using MMFF94s force field model in Molecular Operating Environment (MOE) v.2011.10 software (Chemical Computing Group, Montreal, QC, Canada). Cellular localization of FR47 was predicted using PSORT II, a program that detects sorting signals in proteins and predicts their subcellular localization [16]. Membrane boundaries and protein interactions were obtained from the PPM server and visualized in MOE [17]. Functional insight was obtained by PredictProtein open analysis and looking for conserved SH2 binding domains using DOMPEP [18].

### 2.5. Statistics

PCR products were separated on 1% agarose gel stained with SYBR Green and visualized using the ChemiGenius imaging system (Syngene, Fredrick, MD, USA). To ensure that saturation did not occur, the most dilute cDNA samples that produced visible bands were used for quantification by the GeneTools program. Expression of *α*-*tubulin* was found to be constantly expressed in all tissues and stresses used in this study and was, therefore, chosen as a suitable housekeeping gene to normalize* fr47* transcript expression. Data are expressed relative to control values (with the exception of the development study which was expressed relative to Gosner stages 14–20 [14]) and statistically significant changes in expression were determined by ANOVA with a post hoc Student-Newman-Keuls (SNK) test using SigmaPlot (v.11). All statistical differences are marked according to the results of a post hoc SNK analysis.

## 3. Results

### 3.1. Prediction of FR47 Structure, Localization, and Function

**Figure 1:**
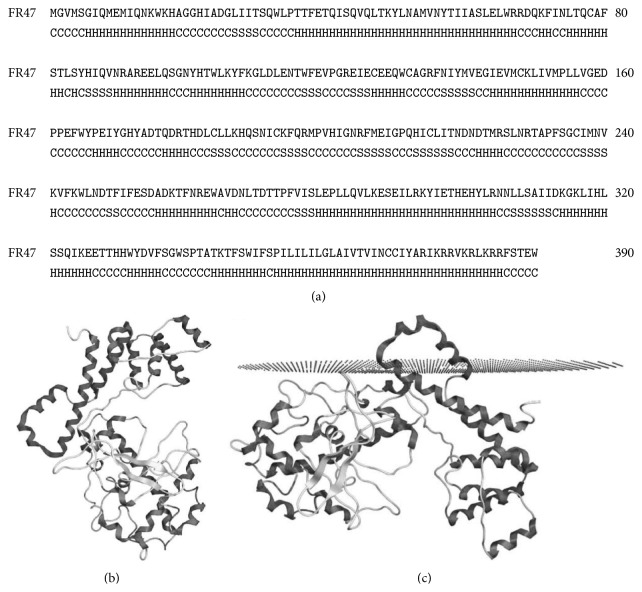
Predicted* de novo* structure of novel freeze responsive protein, FR47. (a) The secondary and (b) 3-dimensional structures of FR47 were determined using I-TASSER webserver, followed by model optimization using MOE. (c) Membrane interactions of FR47 with the intracellular membrane (represented as dots), as predicted using the PPM webserver and visualized with MOE.

**Figure 2:**
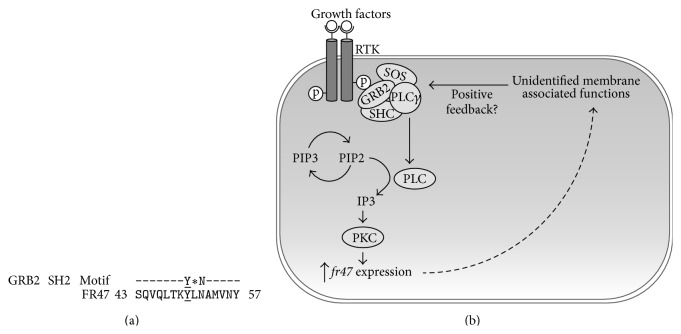
Prediction of SH2 binding motif present in FR47. To elucidate a possible membrane associated function for FR47, DOMPEP was used to identify any possible conserved SH2 binding motifs. (a) Positive identification of the GRB2 SH2 domain phosphotyrosine binding site within the FR47 primary amino acid sequence; “*∗*” indicates hydrophobic amino acids. (b) Hypothesized effect of GRB2-SOS signaling on IP3 production and PKC activation. Increased PKC activity is known to increase* fr47* production and the presence of a GRB2 binding motif in FR47 could suggest that FR47 may create a feedback mechanism to regulate FR47 expression.

Protein structure was predicted from the previously published primary amino acid sequences of FR47 from* R. sylvatica* (GenBank accession AY100690). Following structure prediction using the I-TASSER program (Figures 1(a) and 1(b)), the FR47 protein was predicted to have a high probability of an intracellular localization and was shown to have a high degree of membrane association (Figure 1(c)). As FR47 was predicted to have a high probability of interaction with cell membrane, we used DOMPEP to find any highly conserved SH2 binding motifs as a means of identifying any possible protein interactions that could have a role in signal transduction during the freezing stress. Although the phosphorylation status of FR47 is unknown at this time, DOMPEP analysis revealed a highly conserved motif involved in phosphotyrosine growth factor receptor-bound protein 2 (GRB2) binding at position 50 (SQVQLTKYLNAMVNY) (prediction score: 1.00) (Figure 2). Interestingly, this site was also predicted to be within a protein-binding region by PredictProtein open analysis (SO:0000410).

### 3.2. Response of* fr47* to Freezing

**Figure 3:**
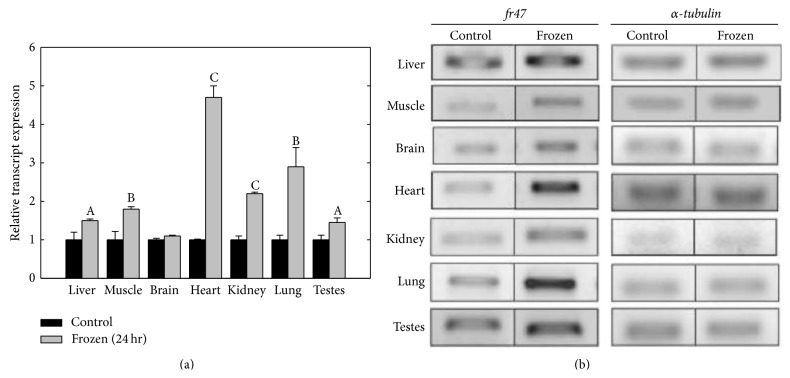
Relative* fr47* transcript expression in response to 24 hr freezing. RT-PCR analysis showing the effects of 24 hr freezing on* fr47* mRNA transcript levels in select tissues of the wood frog. (a) Histogram shows mean values (±SEM, *n* = 4-5 independent determinations) for* fr47*. (b) Representative* fr47* and *α*-*tubulin* bands used for quantification. Data were analyzed using analysis of variance with a post hoc Student-Newman-Keuls test where the letters “A” (*P* < 0.05), “B” (*P* < 0.01), and “C” (*P* < 0.005) represent significant differences between indicated stress and control.

A subsequent multitissue analysis of* fr47* mRNA levels supported previously published patterns of freeze-response in wood frogs.  Figure 3 shows that in response to 24 hr freezing,* fr47* transcript levels significantly increased in the heart by 4.7 ± 0.4-fold when compared to control values (*P* < 0.05). Significantly elevated* fr47* transcript levels were also observed in kidney, lung, and testes after 24 hr freezing (2.0 ± 0.1-, 2.9 ± 0.6-, and 1.5 ± 0.1-fold, resp.) when compared to control values (*P* < 0.05). Brain tissue displayed no change in* fr47* transcript levels between control and 24 hr frozen frogs.

### 3.3. Response of* fr47* to Anoxia and Dehydration

**Figure 4:**
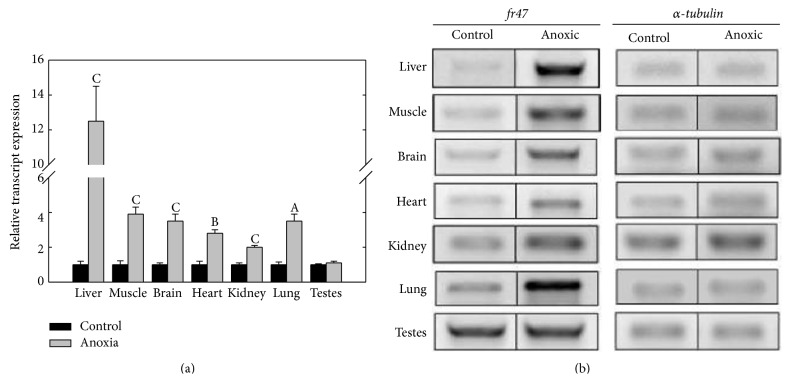
Relative* fr47* transcript expression in response to 24 hr of anoxia. RT-PCR analysis showing the effects of 24 hr anoxia exposure on* fr47* transcript levels in seven tissues of wood frogs. (a) Histogram shows mean values (±SEM, *n* = 4-5 independent determinations) for* fr47*. (b) Representative* fr47* and *α*-*tubulin* bands used for quantification. Other information as in Figure 3.

**Figure 5:**
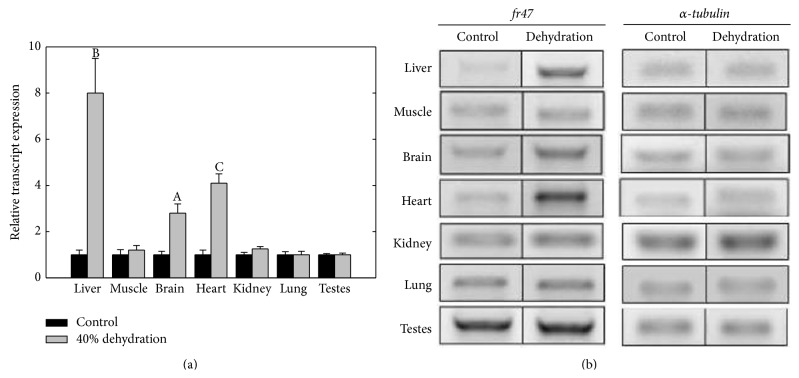
Relative* fr47* transcript expression in response to 40% dehydration. RT-PCR analysis showing the effects of 40% dehydration on* fr47* transcript levels in seven tissues of wood frogs. (a) Histogram shows mean values (±SEM, *n* = 4-5 independent determinations) for* fr47*. (b) Representative* fr47* and *α*-*tubulin* bands used for quantification. Other information as in Figure 3.

To determine the response of* fr47* to anoxia and dehydration, transcript levels were analyzed in frogs exposed to either 24 hr anoxia or 40% dehydration. Compared to control samples,* fr47* transcript levels increased in all tissues with the exception of testes after frogs were exposed to 24 hr anoxia (Figure 4). Liver tissue showed the greatest increase in* fr47* transcript expression, increasing 12.7 ± 2.4-fold higher than control values (*P* < 0.05). Compared to control values, a significant increase in* fr47* transcript expression in response to 24 hr anoxia was also found in skeletal muscle (3.9 ± 0.6-fold), brain (3.1 ± 0.4-fold), lung (2.9 ± 0.6-fold), heart (2.2 ± 0.2-fold), and kidney (1.6 ± 0.1-fold) (*P* < 0.05). Only testes showed no change in* fr47* expression after 24 hr anoxia. Dehydration to 40% of the total body water lost also triggered increased expression of* fr47* in three of the seven tissues tested (Figure 5). Liver, heart, and brain tissues displayed a dehydration-responsive increase by 8.0 ± 1.3-, 3.9 ± 0.3-, and 2.5 ± 0.4-fold when compared to control values, respectively (*P* < 0.05). The remaining four tissues showed no change in* fr47* expression in response to dehydration.

### 3.4. Expression of* fr47* during Wood Frog Development

**Figure 6:**
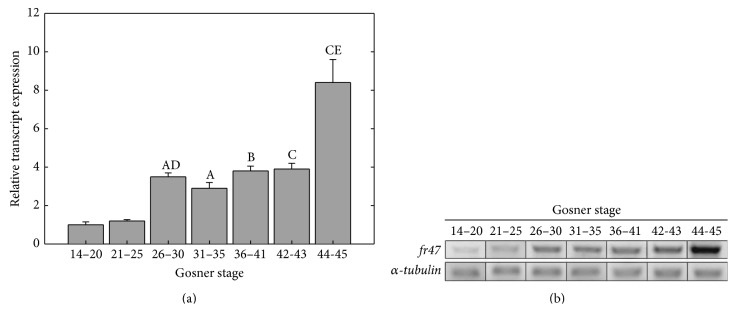
Relative* fr47* transcript expression during stages of tadpole development in wood frogs. (a) Histogram shows mean values (±SEM, *n* = 4-5 independent determinations) for* fr47*. (b) Representative* fr47* and *α*-*tubulin* bands used for quantification. Data was analyzed using analysis of variance with a post hoc Student-Newman-Keuls test where the letters “A,” “B,” and “C” represent significant differences between Gosner stages 14–20 and later stages, whereas “D” and “E” indicate significant differences between the indicated stage and the one preceding it; “A” and “D” indicate *P* < 0.05 and “B” indicates *P* < 0.01, while “C” and “E” indicate *P* < 0.005.

The transcript level for* fr47* was also observed throughout seven stages of tadpole development in the wood frog (Figure 6). Expression gradually increased throughout development, finally resulting in an 8.3 ± 0.9-fold increase in* fr47* transcript expression between Gosner stages 14–20 (where the embryo has a neural fold, used as a starting point) and Gosner stages 44-45 (where the frog is almost fully developed). The increases first became apparent at stages 26–30, where the back limp buds first appearing coincided with a 3.0 ± 0.2-fold increase in* fr47* expression. The level of* fr47* transcription remained fairly consistent over the next three stages of development, with the final stage experiencing greater than 2-fold increase over the previous stage.

## 4. Discussion

To facilitate winter survival, wood frogs have evolved multiple adaptations that help minimize damage and extend viability while in the frozen state. Previous studies have identified three genes that were not only upregulated in response to freezing but also unique to frogs with no known gene homologues [1, 7–9]. While the function and regulation of the two smaller proteins* fr10* and* li16* have been previously explored, the* fr47* gene is the largest and least understood of these three novel freeze responsive genes [11]. As such, this present study extends our knowledge about the regulation and function of* fr47*.

PCR analysis found that* fr47* is expressed in all seven wood frog tissues tested. However, the magnitude of* fr47* transcript increase in response to 24 hr of freezing exposure was dependent on the organ. Freezing increased the expression of* fr47* in six of the seven tissues tested when compared to control conditions. This differs from a previous study that used northern blotting data to show an increase in* fr47* transcripts in liver but no change in brain, heart, lung, kidney, or skeletal muscle tissues in response to freezing [8]. The difference in these findings is likely due to the greater sensitivity of PCR, compared with the northern blotting technique. Overall, these results further confirm that* fr47* is responsive to freezing and expands the tissue-specific regulation of the gene. One hypothesis that could justify the organ-specific expression of* fr47* during freezing is that* fr47* gene expression is linked with the frog's “fight of flight” response.

It is well known that within minutes of freezing initiation (i.e., ice crystal nucleation), glucose production and export from the liver rapidly increase [19]. It has been suggested that glucose synthesis arises from an extreme exaggeration of the “fight or flight” response that increases blood glucose levels in all vertebrates during times of stress [19]. This is supported by the fact that adrenaline-blocking compounds (specifically propranolol) also effectively block glucose synthesis during freezing [19, 20]. In parallel with this “fight or flight” response is the fact that organs known to part of the “fight or flight” are also those displaying significant upregulation in* fr47* during freezing. For example, the physiological responses associated with “fight or flight” include an increase in heart rate, respiration, skeletal muscle activity, a release of sugar molecules from the liver, and a suppressed activity of nonessential organs [21]. Interestingly, the strongest increases in* fr47* transcription upon freezing occurred in the heart, lung, kidney, liver, and skeletal muscle (Figure 3). If a “fight or flight” response is being activated in the frog in response to ice nucleation, these organs would be of paramount importance; as the heart rate increases, kidney will consequentially filter blood more quickly, the breathing rate will increase, muscle will ready itself for action, and glucose will be released from the liver. Importantly, it has been shown that freezing onset triggers an immediate twofold increase in the heart beat of wood frogs that decreases as ice accumulates [22].

It has been discovered that nearly all freeze responsive genes are also responsive to either anoxia or dehydration stresses, both of which are companion stresses of freezing [1]. Our data show that while* fr47* transcript levels are increased after 24 hr anoxia in six of the seven tissues tested, only three tissues showed an increase in transcript levels in response to 40% dehydration (Figures 4 and 5). Previous work by McNally et al. [8] on liver tissue agrees with the present results, showing a strong increase in* fr47* transcription as a result of 24 hr anoxia and to 20% dehydration. It is likely that the increase in* fr47* observed at a much higher level of dehydration (40%) in the present study is compounded by the hypoxia that also develops at high water loss.

To help elucidate a possible functional role for FR47 in stress survival, we first created a* de novo* model of its likely protein structure (Figure 1(a)). Given that previous reports have suggested that the hydrophobic C-terminal of FR47 may represent a possible transmembrane segment, we modeled the interaction between FR47 and the cell membrane (Figure 1(b)). Indeed, our FR47 cell membrane model suggests that FR47 may have a possible involvement in cell membrane associated cellular processes, such as signal transduction. Although the phosphorylation status of FR47 is currently unknown, analysis of phosphotyrosine SH2 binding motifs present on FR47 indicated a perfectly conserved GRB2 binding motif (pY-X-N-X, where X is typically a hydrophobic residue). It is possible that FR47 may have a role in regulating GRB2 signaling during the stressed state. GRB2-SOS signaling is known to regulate phospholipase C (PLC) and the production of inositol 1,4,5-triphosphate (IP3) levels, an intracellular secondary messenger of protein kinase C (PKC) [23]. Interestingly, IP3 levels have been shown to increase significantly during freezing in the wood frog, increasing by 3-fold after 4 hr and by 11-fold after 24 hr of freezing [8, 23]. In this regard, McNally et al. [8] showed that* fr47* transcription in the liver increased in both a dose dependent and time dependent manner in response to phorbol 12-myristate 13-acetate (also known as PMA) stimulated PKC activity. It is possible that the freezing-induced increase in IP3 levels may be influenced through interaction between GRB2 signaling and FR47, mediated through the GRB2 SH2 domain, that could provide a feedback mechanism to regulate* fr47* transcription mediated through PKC activity.

Apart from freezing, the expression of* fr47* was determined at several developmental stages from the embryo to adult frog (Figure 6). Expression of* fr47* increased steadily throughout development until the final Gosner stages 44-45 [14], where the frog has almost fully catabolized its tail. The rise in* fr47* transcripts first became significant at Gosner stages 26–30 (when the back limp buds had appeared) and did not significantly change until the last stage of development, where* fr47* transcription was over 8-fold greater than in earlier stages (Gosner stages 14–20). This is significant as wood frog eggs are typically laid in April when the threat of freezing has largely passed. Moreover, in the present study development from eggs to adults all occurred at room temperature and therefore the animals never experienced cold. This indicates that* fr47* must have an undefined biological role in wood frog development that may also have a critical role to facilitate survival during freezing. Interestingly, Gosner stages 26–30 (where the increase in* fr47* transcripts first became significant) also mark the development and use of the lungs by tadpoles [14].

The results for* fr47* expression in the wood frog indicate a protein whose expression is specific to both development and organ. The data also show that* fr47* must play a role in the developing wood frog; however, this role still remains unclear. Based on the multiorgans patterns of* fr47* gene expression, we now have strong evidence to indicate an important role for FR47 in wood frog stress tolerance that justifies continuing efforts to characterize the protein and identify its cellular function and possible interaction with GRB2 signaling and IP3 synthesis.

## Figures and Tables

**Table 1:** List of primers used in this study.

Primer		Sequence
*fr47 *	Forward	5′-TCCACCAGCTTCTCTGTACC-3′
	Reverse	5′-GAGTCAGGATCTGGAATGGA-3′

*α-tubulin *	Forward	5′-GCCTCATTGTCCACCATGAA-3′
	Reverse	5′-GTGTCGGTACTGGATCTGGC-3′
